# Physician referral is associated with recruitment, motivation, and adherence in an exercise intervention study for older adults

**DOI:** 10.1016/j.jnma.2025.11.002

**Published:** 2025-11-15

**Authors:** Rachel Holden, Amanda N. Szabo-Reed, Amber Watts, Jonathan Clutton, Katrina Finley, Sharon Fitzgerald Wolff, Jeffrey M. Burns

**Affiliations:** KU Alzheimer’s Disease Research Center, Fairway, KS, 66205 USA; Szabo-Reed KU Alzheimer’s Disease Research Center, Fairway, KS, 66205 USA, Department of Internal Medicine, University of Kansas Medical Center, Kansas City, KS, 66160, USA; KU Alzheimer’s Disease Research Center, Fairway, KS, 66205 USA, Department of Psychology, University of Kansas, Lawrence, KS, 66045, USA; KU Alzheimer’s Disease Research Center, Fairway, KS, 66205 USA; KU Alzheimer’s Disease Research Center, Fairway, KS, 66205 USA; Department of Population Health, University of Kansas Medical Center, Kansas City, KS, 66160, USA; KU Alzheimer’s Disease Research Center, Fairway, KS, 66205 USA, Department of Neurology, University of Kansas Medical Center, Kansas City, KS, 66103, USA

**Keywords:** Physician referral, Motivation, Recruitment, Adherence, Exercise, Physical activity, Aging, Older adults

## Abstract

**Objective:**

The LEAP! Rx study was a 12-month randomized controlled trial investigating the effects of supervised exercise and education. This paper is a secondary-data analysis exploring differences in recruitment, motivation, adherence, and retention between physician-referred and self-referred participants.

**Methods:**

219 total healthy, older adult participants (72.79 ± 4.83 years), 118 physician-referred and 101 self-referred, were randomized into a structured exercise and education for Alzheimer’s Disease prevention ((*n* = 110, i.e., LEAP! Rx) or a waitlist control group (*n* = 109). Demographics and patterns in recruitment, motivations for participation, adherence, and retention related to age, gender, race, ethnicity, or education were examined between the physician-referred and self-referred groups.

**Results:**

We observed a significant difference in race, but not sex, between the two referral methods. Specifically, the physician-referred group had a higher proportion of African American participants. Attendance to education classes was similar for both referral types. However, self-referred participants had better adherence to the exercise prescription and were also less likely to withdraw from the study. A higher number of physician-referred participants withdrew due to COVID-19 or were lost to follow-up, likely due to the timing of their recruitment into the study. Motivations for participation were not significantly different by referral source however, a trend with recognition was associated with physician referral and study withdrawal.

**Conclusions:**

Findings suggest that physician referrals can enhance the racial diversity of research participants. The lower adherence in the physician-referred group was likely due to the pandemic’s influence on enrollment, highlighting the need for further research on the relationship between recruitment source, motivations, and adherence.

**Trial registration:**

NCT03253341

## INTRODUCTION

Recruitment of older adults into trials for the prevention or treatment of Alzheimer’s disease and related disease (ADRD) presents unique challenges.^1,2^ Older adults often have multiple health comorbidities that might make them ineligible for trials with restrictive inclusion criteria or complicate their safe participation.^3^ Difficulties with memory, understanding, and decision-making can make it challenging for older adults to provide informed consent and adhere to study protocols.^4^ Limited mobility or lack of transportation can hinder access to research sites.^5^ Older adults may lack social support networks to help them adhere to the requirements of trial participation.^6^ Recruitment bias is also a common issue, as researchers may unintentionally exclude older adults due to concerns about their frailty, cognitive status, or ability to complete the study.^7^ Relatedly, obtaining a diverse and representative sample is a highly cited concern^8^ with clinical trial recruitment in older adult populations.

Strategies for effective recruitment into clinical trials require researchers to reduce barriers to participation (e.g., providing transportation, flexible scheduling, using plain language) and utilize diverse recruitment channels.^9^ Research has specifically shown that physician recommendations, including ones associated with health behavior change, such as exercise adoption, carry weight in motivating clinical trial participation related to physical activity and exercise.^10^ In addition to physician encouragement, understanding facilitators of research participation, namely motives for participation, may improve recruitment and retention of clinical trials. Although some reasons for participating in research may be topic specific, motivations like social engagement, symptom improvement, advancing science, helping others, and compensation have been previously suggested as reasons for participation.^11,12^ However, limited research is available on this topic related to exercise and/or Alzheimer’s disease research and additional research is needed to understand how participant’s motivations for participation can be used to optimize recruitment and retention and how this may intersect with physician referral.^13^

LEAP! Rx (Lifestyle Empowerment for Alzheimer’s Prevention) was a unique program at the University of Kansas Medical Center that combined healthcare expertise with certified personal fitness trainers at designated community gyms to support lifestyle changes. The program provided clinicians with a direct tool for prescribing exercise and a framework individualized for each participant to monitor the program’s success through a randomized, controlled clinical trial. Physicians often encourage patients to exercise without specificity to the type of exercise they should do and a lack of follow up to confirm the patient is achieving their goals and improving their health.^10^ Patients are often unaware of resources and how to use them.^14^ One goal of LEAP! Rx was to support follow up of physical activity recommendations by physicians by connecting patients with community-based exercise resources (i.e., personal trainers at a local gym) to develop healthy and safe exercise habits. This manuscript presents a secondary analysis investigating several of the unique aspect associated with the LEAP! Rx program to evaluate the impact of referral source on recruitment, exercise adherence, retention and motivational difference that may influence outcomes. Understanding how referral source and motivations affects study demographics, adherence, and retention may assist in increasing successful recruitment and retention of populations under-represented in research studies.

## METHODS

### Study overview

The methods and primary outcomes for the LEAP! Rx study^15^ (NCT03253341) have previously been published.^16^ In short, the LEAP! Rx study was a 12-month randomized, controlled trial. The study consisted of two groups: an active intervention group that received structured exercise and education, and a waitlist control group.^16^ The LEAP! Rx program’s man focus was to increase and maintain cardiorespiratory fitness (VO_2peak_) and improve chronic disease risk factors, such as insulin resistance (hemoglobin A1c [HbA1c]), body composition (lean mass, fat mass), and lipid profile (total cholesterol, triglycerides, LDL, and HDL)). These aims were measured at baseline, week 12, and week 52 of the program.^15^

### Recruitment

Recruitment for LEAP! Rx began in 2019, initially using referrals through the electronic medical record (Epic-based Best Practice Advisory (BPA)). This system flagged patients who were potentially eligible for research studies based on eligibility criteria. Physicians could then directly refer these patients for further evaluation. An open referral method was also available, where physicians could insert a code into a patient’s notes to trigger a study referral if they were not identified through the BPA.

In September of 2020, due to low enrollment, the public or self-referral mode was added. This involved placing flyers at community centers, the University of Kansas Medical Center, coffee shops, libraries, other public locations, and other outreach methods including, radio, public presentations, research booths at community events, etc. Self-referrals also often included word-of-mouth recommendations from friends or family. Interested individuals would contact the recruitment team for further evaluation. BPA recruitment continued during this time. Study recruitment ended in February of 2022, with the final follow-up assessments completed in February of 2023. The COVID-19 pandemic influenced the recruitment and enrollment of participants in the study.

### Screening and assessments

Screening for LEAP! Rx involved telephone-based questions to ensure participants met the inclusion and exclusion criteria. Published criteria included being clinician or self-referred, 65 or older, ambulatory, fluent in English, sedentary or under active, and able to get physician approval.^16,17^ Demographic data was collected at screening and confirmed at the baseline visit. The LEAP! Rx study assessments included blood draws, a Dual-Energy X-ray Absorptiometry (DEXA) scan, cardiorespiratory fitness testing, cognitive testing, and various questionnaires and surveys via REDCap.^18,19^ These tests were completed at baseline, week 12, and week 52 of the program. Details related to these outcomes can be found elsewhere.^20^

After baseline testing, randomization was performed using a REDCap module^18,19^ to assign the participant to the immediate start or waitlist group. The immediate start group received a one-year fitness center membership and guidance from a Health Coach. The waitlist group received access to the education program and a gym membership after 12 months.^16^

### LEAP! Rx program description

The LEAP! Rx program^15^ included a 12-week empowerment phase and a 40-week lifestyle phase. The program’s goal was to help participants meet physical activity recommendations of 150 min/week of aerobic exercise (over 3 to 5 days/week) and 2 days/week of resistance exercise.^21^ Monthly Alzheimer’s prevention classes were also offered, covering topics like diet, cognitive engagement, sleep, stress reduction. In partnership with the regional network of YMCAs, participants received a 1-year membership. Participants also received a physical activity monitor (Garmin Vivofit 3, Olathe, KS, USA) to encourage increases in activity, though we did not monitor heart rate or physical activity engagement.

### Program adherence

Exercise data was collected weekly from participants via emails containing a REDCap survey link. Exercise logs documented the study week, date, duration, and type of exercise (aerobic or resistance training), as well as participation in group classes or training sessions. Attendance at monthly education classes was also recorded.

### MOTIVES questionnaire

A subsample of 131 participants completed the MOTIVES questionnaire at baseline. The MOTIVES question was not added until September 2020, it was only available to participants enrolling post-COVID. This survey assessed motivations to participate in research.^22^ It was administered on paper with staff assistance or, during the COVID-19 pandemic, via phone. The survey included sections on interest, opinion, curiosity, enjoyment, helping, incentives, and recognition. Each section had four prompts with responses on a 7-point Likert scale, 1 being the ‘strongly disagree’ option and 7 being strongly agree. An average score and standard deviation was calculated for each section. A second part of the survey asked participants to rank 5 prompts from ‘least important’ to ‘most important’. Only one option could be chosen for each ranking. Appendix 1 contains the MOTIVES questionnaire.

### Data analysis

Differences between physician-referred and self-referred groups for all variables were compared using *t*-test for independent means and chi-square for the categorical variables Correlations were also conducted to examine the relationship between exercise adherence and MOTIVES questionnaire responses. Bonferroni corrections were applied to account for type 1 error from multiple comparisons. Multiple regression was used to future explore the impact of referral source on intervention adherence. Multivariate analysis of variance (MANOVA) were used to explore the association between race/ethnicity and motivations for participation. All analyses were conducted using SAS software (Copyright © 2013 SAS Institute Inc. SAS and all other SAS Institute Inc. product or service names are registered trademarks or trademarks of SAS Institute Inc., Cary, NC, USA.).

## RESULTS

Out of 219 participants 118 were physician-referred and 101 self-referred. Of the 118 physician-referred participants, 59 were in the immediate start group and 51 of the 101 self-referred were in the immediate start group. The remaining participants were part of the waitlist group. Demographics by referral method are presented in Table 1. The total sample was mostly female (81.3 %) with an average age of 72.8 (SD = 4.8). A significant difference in race was found between the two groups (*p* < .001). Participants from historically under-represented groups (HUGs) made up 15.1 % of the total sample. Notably, 23.7 % of physician-referred group were from HUG, compared to 5.0 % of participants of the self-referred group (X^2^ = 14.9, *p* < .001). There were significantly more individuals enrolled using self-referred methods following COVID-19 as compared to physician referrals (*p* < 0.01).

Data on adherence to the exercise program is presented in Table 2. Self-referred participants showed significantly higher adherence to the exercise program than physician-referred participants based on their percent of the prescribed weekly goals (i.e., 150 min and two sessions of resistance training) for aerobic (*p* = .05) and resistance training (*p* = .04). Multiple regression was not significant, indicating that this relationship was independent of HUG and being enrolled post-COVID for both aerobic and resistance training. Self-referred participants were also more likely to complete the intervention and less likely to withdraw or terminate early (87.1 % vs 66.1 %, *p* = .03, see Table 3). A higher number of physician-referred participants withdrew due to COVID-19 concerns and loss to follow-up. Table 4 shows all reasons for withdrawal from the study.

**Figure 1.**
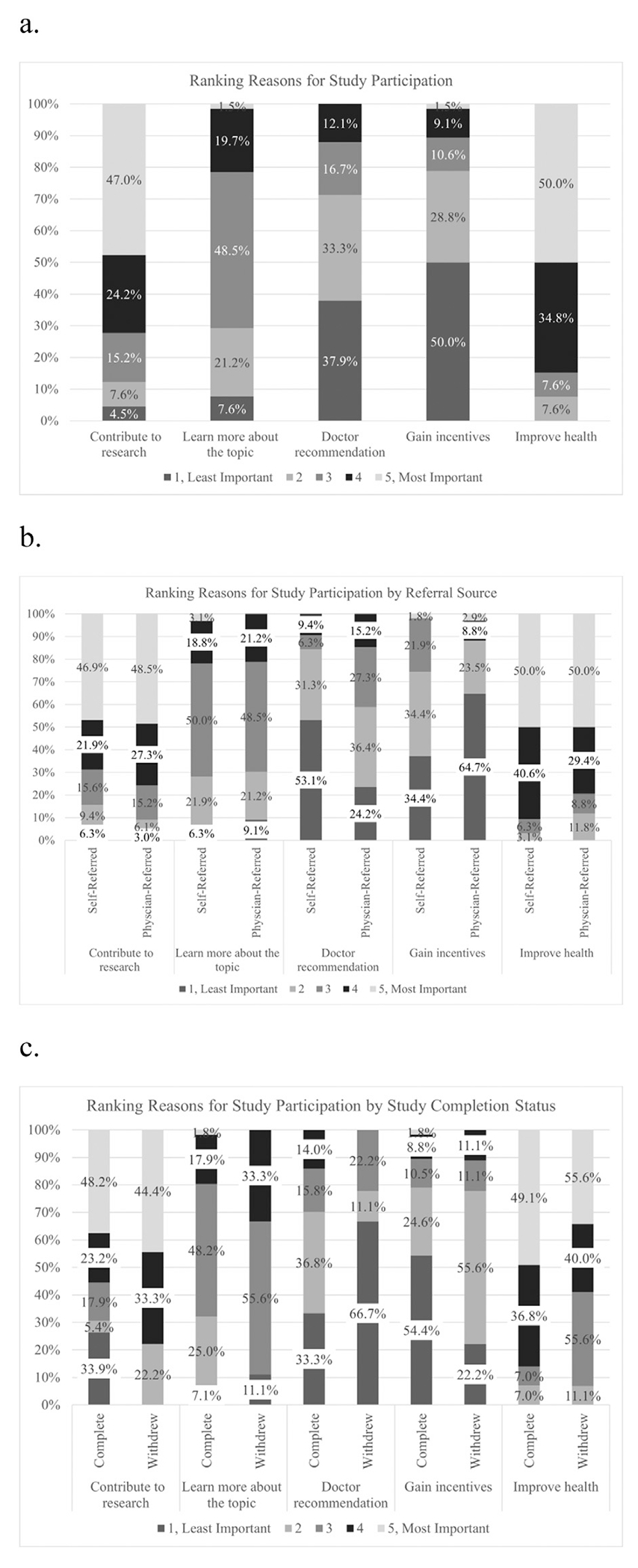
Research participation motivation ranking reasons for all participants and by referral source and study completion status.

For motivations for participating in research (Table 5) between physician referred and self-referred participants show physician referred participants placed a higher value on their opinion of the research being heard during those who were self-referred (*p* < .05). Similarly, those who were physician referred also valued recognition higher than those who were self-referred (*p* < .05). When comparing those who completed the study to those who did not, those in the physician group valued recognition higher than those who were self-referred. In addition improving health and contributing to research were the primary drivers for both groups to participate in the research study, Incentives were ranked as the least important motivator by 50 % of participants (Figure 1). Physician-referred individuals ( *n* = 33) were more likely to rank “gaining incentives” as least important compared to self-referred participants ( *n* = 32) (64.7 % vs. 34.4 %, (X^2^ (4) = 12.09, *p* = .017)). A significant correlation was found between higher exercise adherence and the motivation of “recognition” for those referred by a physician (*p* = .007, Table 6). MANOVA showed no statistical differences in motivations when controlling for HUG status.

## DISCUSSION

The aims of the present study were to investigate differences in sample representativeness, motivations for participation, adherence, and retention for two different recruitment strategies physician-referred versus self-referred participants. The findings point to two main conclusions. First, that self-referred participants had higher rates of adherence than physician-referred participants. Second, that physician-referral led to a more racially and ethnically diverse participant sample. We discuss each of these major findings and possible explanations and implications below.

This secondary data analysis found that self-referred participants in the exercise intervention group adhered to the exercise program at a higher rate than those who were physician-referred. Similarly, self-referred participants were also more likely to complete the intervention and not withdraw or terminate early as compared to physician-referred participants. One explanation for this finding could be that self-referred participants were more internally motivated to make health changes compared to those referred by a physician. To explore this possibility, we investigated whether self-reported motivations for study participation might explain group differences in intervention completion or adherence. We found that individuals who were physician referred placed a higher value on the opinion and recognition of others, such as their physician as compared to those who were self-referred. This was especially true for those who completed the study who were in the physician-referred group.^10^ In addition, improving health (self-focused motives) and contributing to research (altruistic focused motives) were consistently the most frequently reported reasons regardless of whether participants were self-referred of physician referred. Conversely, gaining incentives (like money or a free gym membership), and doctor recommendations were ranked as the least important reasons for joining the study. In addition, there were some differences in how self-referred versus physician-referred, with those where were participants ranked motivations (e.g., self-referred individuals placed less importance on doctor recommendations, and physician-referred individuals cared less about incentives), these differences were not statistically significant. Furthermore, whether participants completed or withdrew from the immediate start group did not correlate with their initial motivations. Finally, there was a significant correlation between a higher exercise adherence and the motivation for recognition for participation for those who were referred to participate by their physician. This suggests that physician follow-up on physical activity recommendations might motivate patients to maintain exercise routines or make other beneficial health behavior changes.

Adherence to exercise was slightly higher in those who were self-referred as compared to physician-referred participants. However, overall adherence to the program was relatively low compared to many of our other community-based exercise studies in older adults.^23,24^ The COVID-19 pandemic may have impacted this finding. All fitness centers closed on March 17, 2020, due to COVID-19 restrictions. During this time, participants in the LEAP! Rx trial had no access to the fitness center until they re-opened on June 1, 2020. These closures only affected physician-referred participants enrolled in the study as self-referral to the study had not yet been opened. During the fitness facility closure, participants were encouraged to exercise at home; however, this was challenging to implement for some participants. Exercise adherence could also have been affected by referral status as well. It may be hypothesized that physician-referred participants may have enrolled in the study because a physician referred them to participate but were not truly interested in exercising.^25,26^ While little research has been done on a self-referral method to exercise, these individuals may have been more self-motivated to exercise. Additional research is warranted on this topic.

Obtaining representative and well-balanced study sample demographic characteristics is challenging for many randomized controlled trials and are an important factor to consider during recruitment. In the LEAP! Rx study, 22.5 % of the physician-referred participants identified as belonging to a HUG, while only 5.0 % of the self-referral population identified as such. Similar studies have found that participants identifying as a HUG would like to participate in research trials, but often do not unless they are told about a specific study or referred to a study by a physician.^27^ This would help explain the difference in our findings for HUG individuals including African American and Hispanic/Latino participants, who were more frequently found in the group with a direct referral through a physician. Similarly, Szabo-Reed et al.^28^ previously reported that professional referrals, including physicians, yielded the greatest percentage of consented individuals in a study evaluating the influence of exercise and intensive vascular pharmacological treatment in older adults.^9^ Overall, the present investigation and prior work indicates that healthcare providers can influence participant recruitment and retention rates, and when providers are engaged, available, and trusted, research participation rates improve.^29,30^

While the numbers for the reason of withdrawals were relatively similar overall amongst the two referral groups, there was a larger number of physician-referred participants withdrawing due to loss of follow-up and COVID-19 concerns. The LEAP! Rx study started before the COVID-19 pandemic and began enrolling only physician-referred participants; thus, these individuals were likely disproportionately affected. By the time self-referrals were added as a method of referral, safer study participation strategies had been implemented (i.e., home exercise and virtual training), which likely led to lower withdrawals due to COVID-19 concerns for the self-referral group. Other studies have also cited such findings during the pandemic.^31^ Withdrawal due to loss to follow-up is more challenging to explain as many studies experience this and for these participants, reasons for ending participation are not given.

The precise impact of physician referrals on exercise participation remains unclear. Some studies suggest that while a doctor’s recommendation might pique a patient’s initial interest, it doesn’t always translate into sustained participation. For example, roughly half of patients referred to exercise don’t follow up on their physical activity, which could explain why many participants are lost during follow-up in exercise studies.^32^ However, a direct referral from a physician can be highly effective. Research involving individuals with multiple sclerosis showed that a direct referral to physical activity was twice as effective in promoting adherence to physical activity guidelines compared to simply providing information.^33^ This study is ral influences a person’s motivation to exercise. Our findings indicate that individuals referred by a physician might participate not just because of the recommendation itself, but also for the recognition from their doctor. More research is needed to fully understand this dynamic. It’s also among the first to explore how the source of refer-important to acknowledge the existing hurdles for physicians. Many face time constraints and a lack of knowledge on how to effectively recommend exercise to their patients, creating a barrier to broader implementation of such referrals.^34–36^

Overall, our results indicate that while physician referral may not be a remedy for ensuring high rates of adherence in exercise interventions, it is a particularly effective strategy for enhancing the racial and ethnic diversity of participant samples. For researchers interested in recruiting historically under-represented groups, a direct referral from a healthcare provider appears to be a powerful and trusted mechanism. Therefore, we would argue that the most robust recruitment strategy for future randomized controlled trials is not to simply choose one method over another, but to design a ’hybrid’ approach that intentionally leverages the strengths of both. For example, a future recruitment plan could combine active physician outreach to build a diverse participant pool with community-based self-referral campaigns aimed at individuals with a higher degree of self-motivation. This balanced approach would address the distinct challenges of both sample representativeness and participant retention.

### Limitations

The most notable limitation to consider is the timing of this study. Not only did we implement a new recruitment method following the start of the pandemic, but adherence and withdrawal from the study due to the pandemic also impacted study outcomes. Unfortunately, all these factors cannot be accounted for statistically, and we can only speculate on how the results might have differed at a different point in time. Another limitation of this study is that participants self-reported their exercise minutes and activities. The study team could not access the fitness tracker or utilize another device that may have provided more accurate information. In addition, the education classes also began as in-person meetings, but they moved to online video meetings post-pandemic. This change did not seem to affect attendance, but the method of class instruction did change. Finally, this study is a secondary data analysis of the LEAP! Rx trial and therefore it was not powered to test these associations. Thus, future powered trials are warranted to do so.

## CONCLUSION

In conclusion, our findings indicate that physician referrals are a particularly valuable strategy for enhancing the racial and ethnic diversity of participant samples in research studies. While this method may not guarantee high rates of adherence, its effectiveness in reaching under-represented groups highlights its unique value. Therefore, we conclude that the most effective recruitment model for exercise-based interventions would be a multi-pronged approach that leverages physician referrals to promote diversity, while also including a strategic self-referral component to capture individuals who may be more intrinsically motivated to adhere to the program. This combined strategy will be crucial for conducting research that is both representative and successful in achieving its intended health outcomes.

## Supplementary Material

1

Supplementary material associated with this article can be found, in the online version, at doi:10.1016/j.jnma.2025.11.002.

## Figures and Tables

**Table 1.** Demographics by Referral Method.

	Physician-Referred	Self-Referred	Total Sample
Referral Method (n)	118	101	219
Age, years (Mean, SD)	72.79, 4.83	71.86, 4.82	72.36, 4.84
Sex (n (%))			
Female	98 (83.0 %)	80 (79.2 %)	178 (81.3 %)
Male	20 (17.0 %)	21 (20.8 %)	41 (18.7 %)
Race (n (%))			
White	90 (76.3 %)	96 (95.0 %)	186 (84.9 %)
Historically Underrepresented Groups	28 (23.7 %) *	5 (5.0 %) *	33 (15.1 %)
Ethnicity (n (%))			
Hispanic/Latino	2 (1.7 %)	4 (4.0 %)	6 (2.7 %)
Not Hispanic/Latino	40 (33.9 %)	89 (88.1 %)	129 (58.9 %)
Unknown	76 (64.4 %)	8 (7.9 %)	84 (38.4 %)
Education, years (Mean, SD)	15.75, 2.54	16.24, 2.05	15.97, 2.34
Study Group (n (%))			
Intervention	59 (50.0 %)	51 (50.5 %)	110 (50.2 %)
Control	59 (50.0 %)	50 (49.5 %)	109 (49.8 %)
Enrolled Post COVID (n (%))			
Intervention	27 (34.6 %)*	51 (65.4 %)*	78 (50.3 %)
Control	27 (35.1 %)*	50 (64.9 %)*	77 (49.7 %)

* = indicates p-value < 0.05.

**Table 2.** Exercise Adherence by Referral Method (Immediate Start Only).

	Physician Referred (*n* = 59)	Self-Referred (*n* = 51)
Aerobic Exercise Adherence (%)	60.56 (47.06) %*	76.27 (38.12) %
Resistance Exercise Adherence (mean, SD, %)	58.91 (39.59) %*	77.73 (58.07) %
Percentage of Education Classes Attended (mean % (SD))	26.10 (29.20) %	26.47 (31.78) %

* = indicates p-value ≤0.05.

**Table 3.** Completion of Study by Referral Method.

	Physician Referred (*n* = 118)	Self-Referred (*n* = 101)
Completed Intervention (n (%))	78 (66.1 %) *	88 (87.1 %)
Intervention	39	44
Control	39	44
Withdrew/Terminated Early (n (%))	40 (33.9 %) *	13 (12.9 %)
Intervention	20	7
Control	20	6

* = indicates p-value ≤0.05.

**Table 4.** Reasons for Termination or Withdrawal by Referral Method.

	Physician Referred (*n* = 40)	Self-Referred (*n* = 13)
Serious Adverse Event (SAE) (n (%))		
Intervention	1 (2.5 %)	0 (0.0 %)
Control	0 (0.0 %)	0 (0.0 %)
Loss to Follow-Up (n (%))		
Intervention	8 (20.0 %)	2 (15.4 %)
Control	4 (10.0 %)	1 (7.7 %)
COVID-19 Concerns (n (%))		
Intervention	2 (5.0 %)	0 (0.0 %)
Control	2 (5.0 %)	0 (0.0 %)
Health Problems (n (%))		
Intervention	5 (12.5 %)	2 (15.4 %)
Control	1 (2.5 %)	2 (15.4 %)
Change in Circumstances (n (%))		
Intervention	4 (10.0 %)	2 (15.4 %)
Control	3 (7.5 %)	2 (15.4 %)
Problem with Waitlist Group (n (%))		
Intervention	0 (0.0 %)	0 (0.0 %)
Control	4 (10.0 %)	1 (7.7 %)
Uncomfortable with Testing Procedures (n (%))		
Intervention	0 (0.0 %)	0 (0.0 %)
Control	4 (10.0 %)	0 (0.0 %)
Other (n (%))		
Intervention	0 (0.0 %)	1 (7.7 %)
Control	2 (5.0 %)	0 (0.0 %)

**Table 5.** Mean Motives Survey Responses for Immediate Start Group.

	Physician-Referred Avg (*n* = 33)	Self-Referred Avg (*n* = 32)	Total Sample Avg (*n* = 65)
Interest			
All	6.41 ± 0.70	6.53 ± 0.91	6.35 ± 0.82
Complete	6.37 ± 0.09	6.26 ± 0.70	6.32 ± 0.60
Withdrew	6.50 ± 0.50	6.37 ± 0.84	6.42 ± 0.72
Opinion			
All	5.24 ± 1.08	4.77 ± 1.40	5.01 ± 1.27*
Complete	6.37 ± 0.51	4.68 ± 1.21*	4.99 ± 1.10
Withdrew	5.25 ± 0.90	5.12 ± 0.99	5.12 ± 0.91
Curiosity			
All	6.07 ± 0.78	6.00 ± 0.89	6.04 ± 0.83
Complete	6.14 ± 0.64	5.98 ± 0.67	6.01 ± 0.65
Withdrew	6.42 ± 0.38	6.08 ± 1.03	6.19 ± 0.85
Enjoyment			
All	5.28 ± 0.96	5.17 ± 1.24	5.23 ± 1.10
Complete	5.17 ± 0.84	5.19 ± 1.02	5.17 ± 0.92
Withdrew	6.00 ± 1.00	5.08 ± 1.24	5.39 ± 1.19
Help			
All	6.51 ± 0.66	6.40 ± 1.13	6.45 ± 0.92
Complete	6.42 ± 0.60	6.38 ± 1.07	6.40 ± 0.84
Withdrew	6.91 ± 0.92	6.45 ± 0.93	6.61 ± 0.77
Incentive			
All	4.95 ± 1.51	5.03 ± 1.97	4.99 ± 1.74
Complete	4.90 ± 1.21	4.98 ± 1.67	4.94 ± 1.43
Withdrew	5.50 ± 1.00	5.25 ± 2.14	5.33 ± 1.77
Recognition			
All	2.53 ± 1.29	2.97 ± 1.57	2.75 ± 1.45*
Complete	2.59 ± 0.93	2.74 ± 1.18	2.66 ± 1.05
Withdrew	1.75 ± 1.29	3.96 ± 1.16	3.22 ± 1.57
Obligation			
All	4.42 ± 1.56	4.41 ± 1.50	4.42 ± 1.54
Complete	4.46 ± 1.23	4.29 ± 1.02	4.39 ± 1.13
Withdrew	4.42 ± 1.18	4.92 ± 0.83	4.75 ± 0.92

* p < .*05*.

**Table 6.** Correlation Between Exercise and MOTIVES Responses.

	Physician-Referred Avg (*n* = 33)	Self-Referred Avg (*n* = 32)	Total Sample Avg (*n* = 65)
Interest	−0.077	−0.104	−0.083
Opinion	−0.236	0.040	−0.050
Curiosity	−0.147	−0.037	−0.059
Enjoyment	−0.108	0.098	0.028
Help	−0.010	−0.035	−0.048
Incentive	−0.204	0.164	0.038
Recognition	0.211*	−0.013	0.051
Obligation	−0.084	−0.193	−0.141

* p ≤.*01*.
