# Supplementary figures and images for: Physician referral is associated with recruitment, motivation, and adherence in an exercise intervention study for older adults
Source: J Natl Med Assoc. Author manuscript; Available in PMC 2026 Jul 17. (PMC13377785; doi:10.1016/j.jnma.2025.11.002)

Appendix 1.
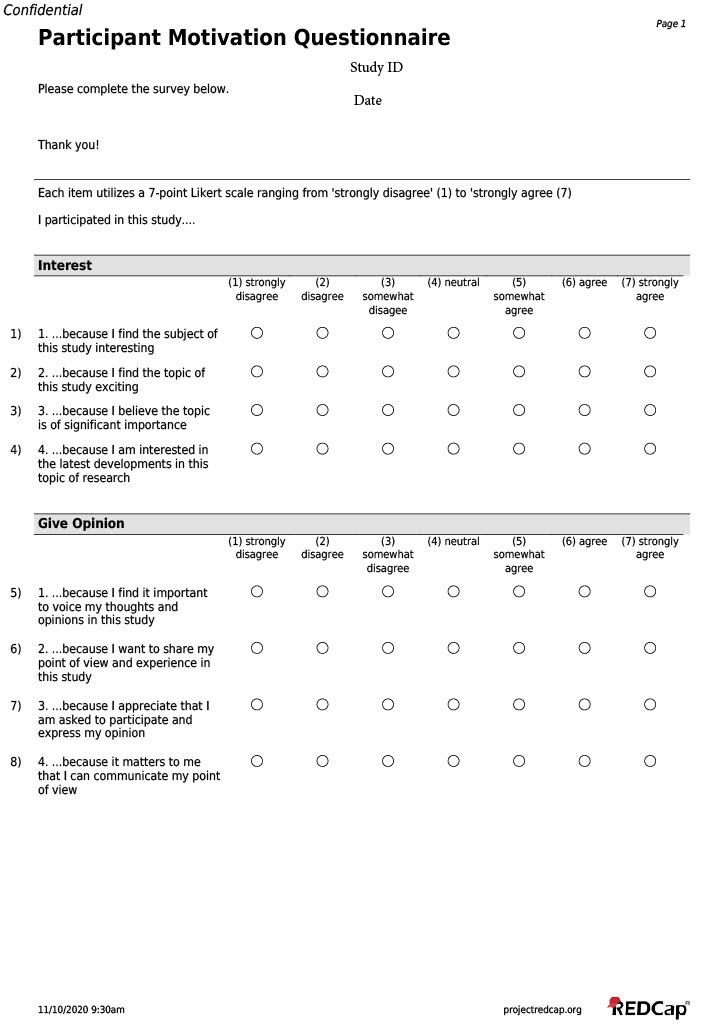


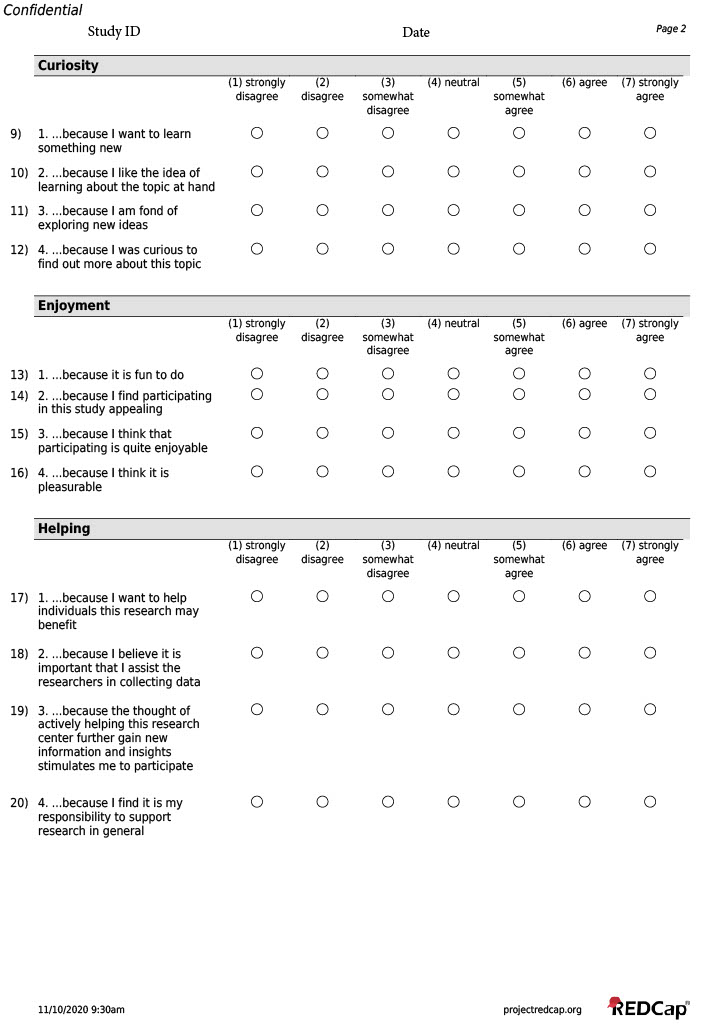


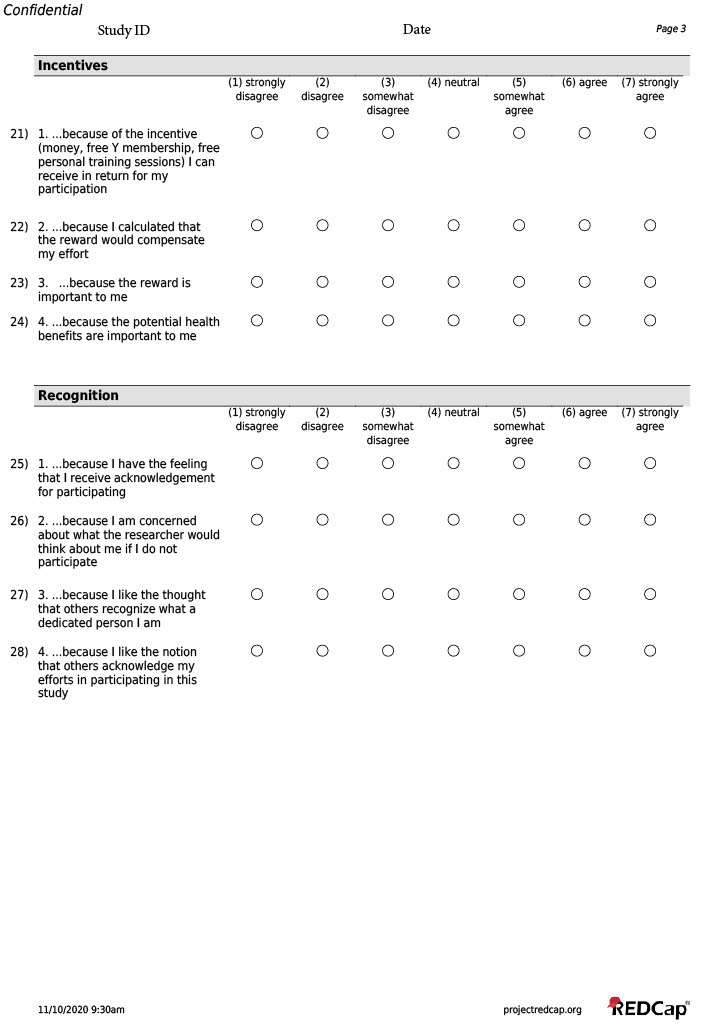


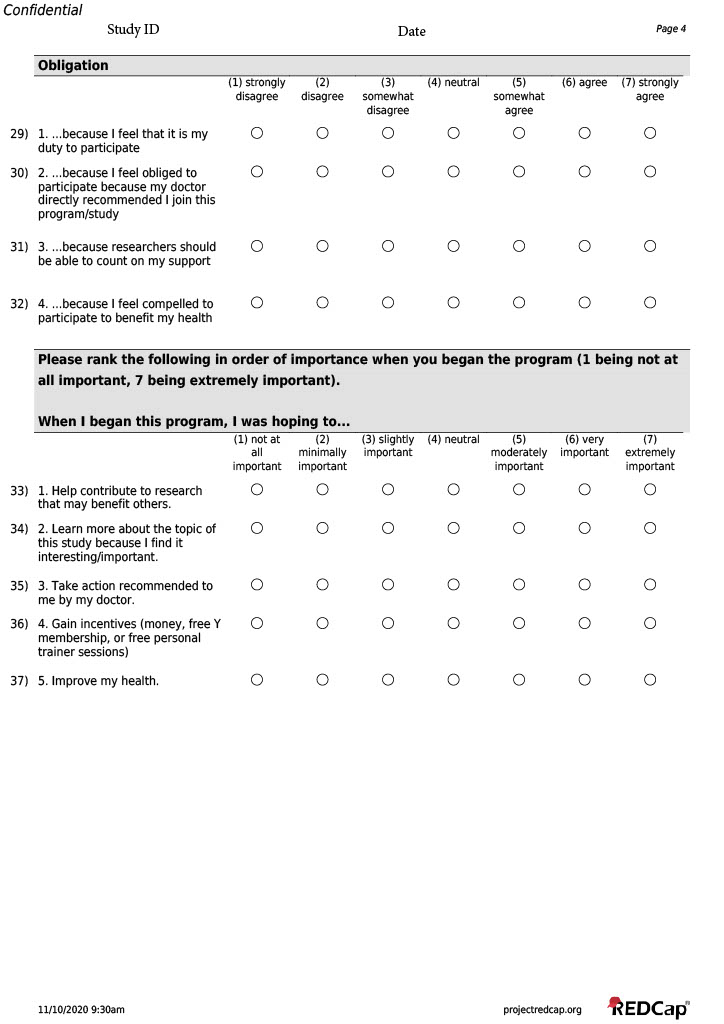

Supplement: 1 [file NIHMS2186301-supplement-1.docx]
